# The ideological divide in confidence in science and participation in medical research

**DOI:** 10.1038/s41598-021-82516-6

**Published:** 2021-02-04

**Authors:** Matthew Gabel, Jonathan Gooblar, Catherine M. Roe, John C. Morris

**Affiliations:** 1 Washington University in St. Louis, St. Louis, USA; 2 Stanford University School of Medicine, Stanford, USA

**Keywords:** Medical research, Clinical trial design

## Abstract

In the United States, the wide ideological divergence in public confidence in science poses a potentially significant problem for the scientific enterprise. We examine the behavioral consequences of this ideological divide for Americans’ contributions to medical research. Based on a mass survey of American adults, we find that engagement in a wide range of medical research activities is a function of a latent propensity to participate. The propensity is systematically higher among liberals than among conservatives. A substantial part of this ideological divide is due to conservative Americans’ lower confidence in science. These findings raise important issues for the recruitment of subjects for medical studies and the generalizability of results from such studies.

## Introduction

In the United States, the wide ideological divergence in public confidence in science poses a potentially significant problem for the scientific enterprise. Americans with a conservative political ideology gradually lost confidence in the scientific community over the last thirty years^1^. This decline resulted from several sources of conflict between science and conservative values. Beginning in the 1970s, the “new right” emerged to challenge science based on conservative interests related to religion, traditionalism, and government regulation^1^. This challenge to science was successfully articulated through conservative media and partisan political institutions^1–4^. The current wide ideological divide between conservatives and liberals appears to be stable^1,5^. Given the number and political importance of conservatives, and the relative stability of Americans’ ideological commitments, this divide could portend significant obstacles for the practice, advance, and influence of science in the United States^6^.

In this study, we examine the behavioral consequences of this ideological divide for Americans’ contributions to medical research. The ideological divide should be relevant for how Americans engage medical research because that vast majority of Americans consider the field of medicine as central to the scientific enterprise^7^. Consequently, we hypothesize that conservatives, due to their lower confidence in science, have a smaller propensity to participate in medical research than do liberals.

Public participation is critical for a wide range of medical activities. Perhaps most directly, longitudinal cohort studies depend on recruiting large numbers of study subjects. Such studies are an important tool for understanding a variety of diseases and are increasingly attractive for identifying the interaction of genetics, environment, and lifestyle^8^. But public contributions comprise many levels of engagement, ranging from onerous and long-term commitments as study participants to sporadic or single events, such as participating in a medical fundraiser.

An ideological divide in such participation could undermine both the execution and quality of medical research. Given the uneven geographic distribution of political ideology, it could pose distinct challenges for local, regional, and state strategies for recruiting and retaining study participants and developing and maintaining political support for medical research^9,10^. An ideological divide could also threaten the generalizability of medical studies, since important types of health behaviors (e.g., smoking) vary with Americans’ political ideology^11–14^.

We tested our hypothesis with a mass survey of American adults. Our analysis includes a range of medical research activities, including prominent and demanding types of participation—e.g., clinical trials. Because these more demanding forms of participation typically exclude younger adults, we focus on older American (at least 45 years old). This allows us to examine participation by a subset of Americans highly relevant to medical research^15–17^. We find strong evidence that Americans approach a diverse set of opportunities to contribute to medical research based on a general aversion or inclination to participate. Consistent with our expectation, we then show that confidence in science mediates the effect of political ideology on the propensity to participate, with conservatives less likely to participate than liberals due, in part, to their lower confidence in science.

These results are consistent with a recent finding that Americans’ willingness to participate in a hypothetical longitudinal study of Alzheimer Disease (AD) was lower among conservatives as a result of their weaker confidence in science^18^. That study cautioned that its finding might not generalize to other areas of medical research. Our analysis addressed this issue directly, as we included the same hypothetical AD study survey question in our analysis. Finally, our study provides evidence pertinent to the recruitment and retention of study participants.

## Results

To test our hypothesis that participation in medical research activities decreases with conservative political ideology and that this effect is mediated through their lower confidence in science, we analyzed survey data from the July 2014 and September 2015 waves of the American Panel Survey (TAPS). TAPS is a monthly on-line panel survey of a probability-based representative sample of American adults conducted by Knowledge Networks. The TAPS survey was reviewed and deemed exempt by the Washington University Institutional Review Board.

### Measure of propensity to participate in medical research

The survey asked four questions about past participation in medical research related activities: a clinical trial for a drug, a long-term observational study, a fundraiser for medical research, and blood donation. It also included a prospective hypothetical question about one’s willingness to donate an organ upon death. Finally, the survey included the same question about participation in a hypothetical longitudinal AD study analyzed in a previous study^18^. That survey question was asked in a 2014 wave of the same TAPS panel survey. For all six questions, we recoded responses as (1) if the respondent participated or was willing to participate, and (0) otherwise^19^. Descriptive statistics are presented in Table 1.

**Table 1:** Descriptive statistics for TAPS survey respondents at least 45 years old.

Variable	N	%	Mean	Standard deviation
Participated in clinical trial	1117	6.18	–	–
Participated in medical fundraiser	1117	49.23	–	–
Participated in long-term medical study	1123	8.10	–	–
Donated blood	1123	68.21	–	–
Willing to donate organ	1119	71.04	–	–
Willing to participate in Alzheimer Disease study	995	17.39	–	–
African American	1123	7.03	–	–
Hispanic	1123	7.22		
Female	1121	48.62	–	–
Resident of metropolitan area	1123	84.95	–	–
Education (15 categories)	1116	–	11.28	1.86
Political ideology (conservative-liberal)	1107	–	2.81	1.78
Propensity to participate	983	–	0.00	0.68
Confidence in science	995	–	1.70	0.83
General confidence in institutions	974	–	0.00	0.88
Age (4 categories)	1123	–	3.55	0.49

We expect answers to each of these questions to reflect one’s propensity to participate in medical research. But responses to any one question likely provide a noisy measure of one’s general propensity. These survey questions vary in the type of medical research activity and in the difficulty of participation. Participation in a clinical trial, a long-term medical study or the hypothetical AD study requires significantly greater commitment than a blood donation. Also, participation in each activity may reflect activity-specific considerations. For example, one may be more willing to participate in the AD study if one has a parent who has died from AD. For these reasons, we estimate an Item Response Theory (IRT) measurement model. The IRT model generates a measure of the latent propensity to participate in medical research that underlies responses across all forms of participation. In addition, the model allows us to validate the measure of propensity to participate by examining how the individual survey questions relate to the latent dimension.

We restricted our analysis to respondents 45 years of age or older, as younger respondents would generally have been ineligible to participate in the long-term studies, the hypothetical AD study, and the clinical trials described in the survey questions. For example, most clinical trials for drugs involve cancer, AD, cardiovascular diseases, obesity, and diabetes and recruit participants with symptoms or the disease^20^. Because the very low incidence of these diseases in younger adults, these studies typically include patients in their mid-40 s and older^21,22^. For example, a recent review of clinical trials indicates that the vast majority (95%) of participants in studies of common diseases were at least in their early 40s: lung cancer (age 40 and older), atherosclerosis (age 46 and older), coronary dysrhythmias (age 49 and older), and prostate cancer (age 56 and older)^20,21^. Similarly, AD studies typically exclude younger participants, as the earliest symptoms typically appear between 46 and 48 years old^23,24^. For that reason, the long-term AD studies that are most similar to the hypothetical AD study in our survey question have a minimum participation age of 45 years old^25^.

Given these age considerations regarding the medical research activities in our study, we chose to include only respondents that were at least 45 years old. However, to ensure the results are robust to other plausible age limits, we conducted separate analyses with the inclusion of all respondents over 40 years old and over 50 years old. Further description of the survey items and the coding protocol is presented in the methods section. Note the results presented below are robust to alternative coding, sampling weights, and corrections for missing data (see Supplementary Table S1 online).

We use this battery of six questions to evaluate whether responses about distinct forms of participation reflect a common underlying (latent) commitment to medical research. A polychoric factor analysis of the six items clearly indicates that a single latent dimension structures participation. For all three age inclusion rules, the eigenvalue for the first factor was greater than 1.59 and the eigenvalue for the second factors was less than 0.52. Furthermore, the items all loaded on the factor in the same direction, indicating that participation in one activity was positively associated with participation in the other activities.

To further validate the latent dimension and to generate a score for each respondent on that dimension, we estimated an Item Response Theory (IRT) model. The IRT model is designed to use the survey items to distinguish among respondents based on a general propensity to participate. Formally, we estimate the following logistic IRT model of a respondent’s decision to participate in each research activity:

$$\Pr {(}Y_{i,j} = 1 {|} \alpha_{i} , \beta_{i} ,\theta_{j} ) = \frac{{\exp \left( {\alpha_{i} \theta_{j} + \beta_{i} } \right)}}{{1 + {\text{exp}}\left( {\alpha_{i} \theta_{j} + \beta_{i} } \right)}}$$

where *i* indexes the six items (types of participation in medical research), *j* indexes the individual respondents, *Y*_*i,j*_ represents the survey response about participation in each item for each respondent, θ_*j*_ represents the latent trait level (i.e., propensity to participate) for each respondent, ${\beta }_{i}$ represents the difficulty of item *i*, and α_*i*_ represents the discrimination of item *i*.

The difficulty parameter ${\beta }_{i}$ indicates how well an item differentiates among respondents on the latent dimension. An item that requires a greater commitment to medical research should have a higher difficulty parameter than an item that is less demanding. If the latent dimension is capturing respondents’ propensity to participate, we would expect higher difficultly estimates for the items corresponding to long-term studies, clinical trials, and the hypothetical AD study than the other forms of participation. The discrimination parameter ${\alpha }_{i}$ distinguishes items by how strongly they are associated with the latent dimension. Variation in each item is assumed to reflect both item-specific (idiosyncratic) sources and the latent commitment to medical research participation. Items with higher discrimination scores are more strongly a function of respondents’ general propensity to participate.

**Figure 1:**
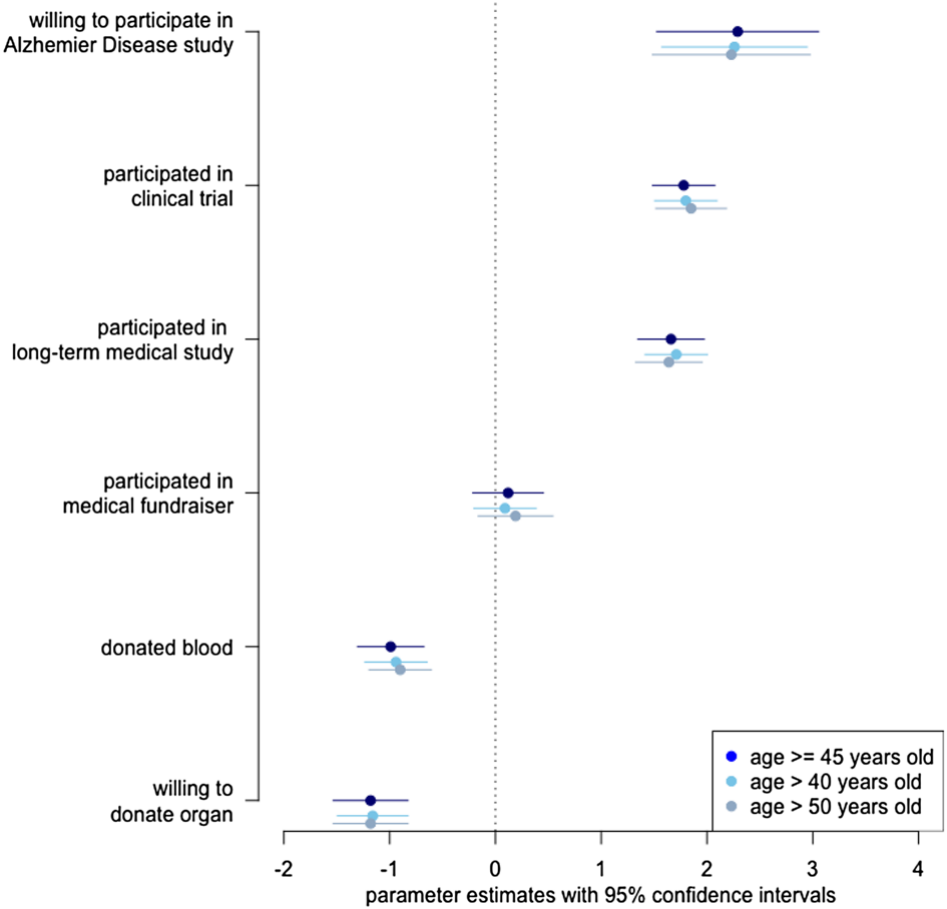
Difficultly parameters from IRT model of participation in medical research.

Figure 1 presents the difficulty parameter estimates for respondents at least 45 years old, and for respondents based on the other two age limits. Consistent with expectations, the highest difficulty scores are for the items with the most demanding participation: a clinical trial of a drug, a long-term medical study, and the longitudinal AD study. Thus, even respondents with relatively high propensity to participate in medical research would find it difficult to agree to these activities. In contrast, donating blood and agreeing to donate an organ upon death have much lower difficulty scores. Thus, the estimated latent dimension captures key substantive distinctions associated with a propensity to participate in medical research.

**Figure 2:**
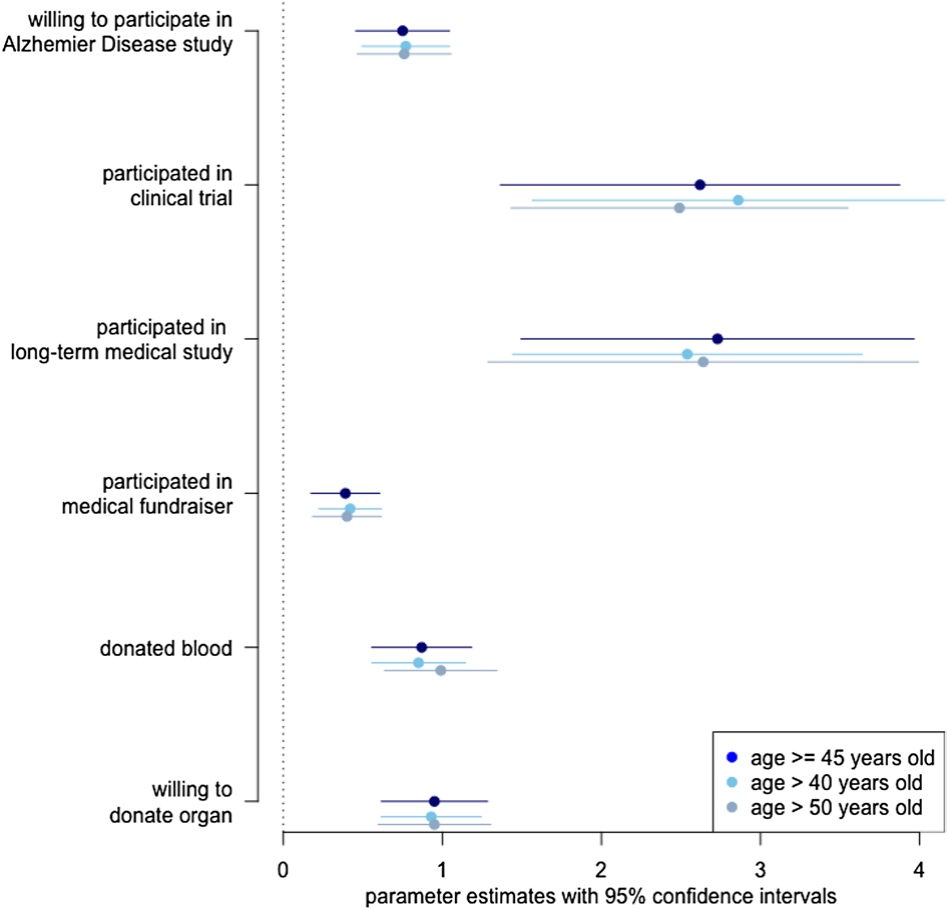
Discrimination parameters from IRT model of participation in medical research.

Figure 2 presents the discrimination parameter estimates for the three age groups of respondents. The discrimination parameters indicate that responses to the questions about clinical trial and long-term study participation are more informative about a respondent’s general propensity to participate than are responses to the other types of activities. The low discrimination parameter for past participation in fundraisers implies that variation in participation was due more to idiosyncratic features of the activity (e.g., the type or location of the event) than were responses to other items. Similarly, the discrimination parameter for the AD study item is relatively low, indicating that the results from the related AD study may not generalize to the general propensity to participate^18^.

### Mediation analysis of political ideology, confidence in science, and the propensity to participate

Based on the IRT model, we generated scores on the latent variable ${\theta }_{j}$ for each respondent. The resulting variable, *Propensity to Participate*, has a mean of zero and standard deviation of 0.67. We then estimated the effect of a respondent’s political ideology—both direct and mediated through confidence in science—on her propensity to participate. Recall our expectation is that, relative to liberals, more conservative respondents will be have a lower propensity to participate in medical research and that this effect should be at least partially mediated through conservatives’ lower confidence in science.

We employed conventional measures of political ideology and confidence in science used in mass surveys of Americans. We measured political ideology based on respondents’ self-placement on a 7-point ideological scale ranging from “very conservative” to “very liberal.” This is a common survey measure of Americans’ political ideology and has been validated by past studies^6,26,27^. We measured respondents’ confidence in science by asking how much confidence they had in the leadership of the scientific community. The 4-point likert response scale ranges from a “great deal of confidence” to “very little confidence.” Research has demonstrated its validity in surveys of the American public and in the study of the ideological divide in confidence in science^1,7,28,29^.

The mediation analysis is based on two ordinary least squares regressions:

1 $$\begin{array}{*{20}c} {Confidence\;in\;Science_{i} = \beta_{0} + \beta_{1} *Ideology_{i} + \gamma *Z_{i} + \varepsilon_{i} } \\ \end{array}$$

2 $$\begin{array}{*{20}c} {Propensity_{i} = \beta_{2} + \beta_{3} *Ideology_{i} + \beta_{4} *Confidence\;in\;Science_{i} + \gamma *Z_{i} + \varepsilon_{i} } \\ \end{array}$$

where *i* indexes individual survey respondents, $Z$ indicates a vector of control variables described in the Methods section, and $\varepsilon_{i}$ is the error term. The product ${\beta }_{1}$*${\beta }_{4}$ represents the indirect or mediated effect of ideology on the propensity to participate.

Table 2 presents the regression results for these two equations for the set of respondents 45 years of age or older, and for the two alternative age limits. The evidence from all three sets of respondents is consistent with expectations. Political ideology positively predicts confidence in science. As ideology becomes more liberal (more conservative), confidence in science increases (decreases). Confidence in science had a positive and statistically significant effect on propensity to participate and mediates the effect of ideology. Finally, ideology has a direct positive effect on propensity to participate.

**Table 2:** Regression results for mediation analysis based on different age subsets of respondents.

	Respondents at least 45 years old		Respondents over 40 years old		Respondents over 50 years old	
	(1)	(2)	(1)	(2)	(1)	(2)
Political Ideology	0.094**	0.031*	0.104**	0.031*	0.092**	0.034*
	(0.014)	(0.013)	(0.014)	(0.012)	(0.015)	(0.013)
Confidence in Science	–	0.090**	–	0.088**	–	0.100**
		(0.032)		(0.029)		(0.034)
Confidence in Institutions	0.351**	0.007	0.338**	0.021	0.344**	0.008
	(0.028)	(0.026)	(0.026)	(0.024)	(0.029)	(0.028)
Age	0.151**	0.099*	0.052	0.102**	0.130**	0.133**
	(0.046)	(0.043)	(0.035)	(0.030)	(0.048)	(0.044)
Female	0.151**	0.040	0.184**	0.051	0.132**	.041
	(0.046)	(0.044)	(0.044)	(0.041)	(0.048)	(0.046)
Education	0.081**	0.055**	0.085**	0.054**	0.077**	0.055**
	(0.013)	(0.012)	(0.013)	(0.011)	(0.014)	(0.013)
African American	− 0.417**	− 0.287**	− 0.422**	− 0.241**	− 0.419**	− 0.280**
	(0.101)	(0.071)	(0.094)	(0.068)	(0.107)	(0.076)
Hispanic	− 0.138	− 0.131	− 0.117	− 0.196**	− 0.043	− 0.224**
	(0.101)	(0.086)	(0.093)	(0.072)	(0.106)	(0.087)
Metropolitan resident	0.191**	0.127*	0.177**	0.107	0.205**	0.116
	(0.067)	(0.059)	(0.064)	(0.055)	(0.070)	(0.062)
R^2^	0.30	0.10	0.29	0.11	0.28	0.11
N	932		1032		850	
% of Effect of Mediated	22%		23%		22%	
	(95% CI: 13%–54%)		(95% CI: 14%–52%)		(95% CI: 13%–50%)	

Each column presents ordinary least squares unstandardized regression coefficients with robust standard errors in parentheses. Column (1) corresponds to Eq. (1) of the mediation analysis (*Confidence in Science* is the dependent variable). Column (2) corresponds to Eq. (2) in the mediation analysis (*Propensity to Participa*te is the dependent variable). *p < 0.05; **p < 0.01, two-tailed test.

**Figure 3:**
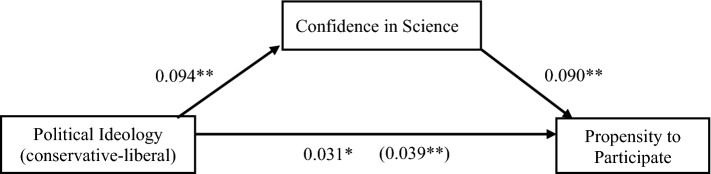
The effect of political ideology on propensity to participate in medical research partially mediated by confidence in science. The values in the figure represent standardized regression coefficients. The value in parentheses is for the total effect of political ideology, which was estimated in a regression without confidence in science. All regressions included controls for confidence in institutions, gender, residence in metropolitan area, age, education, African American ethnicity, and Hispanic ethnicity. **p < 0.01, *p < 0.05, two-tailed test.

Figure 3 presents the path diagram of the direct and mediated effect of political ideology on willingness to participate, based on the set of respondents 45 years or older. A change from very conservative to very liberal is associated with an adjusted average causal mediation effect of 0.05 (95% CI 0.01 to 0.09), the average direct effect is 0.18 (95% CI 0.03–0.33), and the total effect is 0.23 (95% CI 0.09–0.39). 22 per cent (95% CI 13%–54%) of the effect of ideology was mediated by confidence in science.

## Discussion

Our results indicate that, for Americans of sufficient age to participate in most forms of medical activities (i.e., at least 45 years of age), one’s engagement in a range of activities that advance medical research reflects one’s general propensity to participate. We show that this propensity is directly and indirectly related to Americans’ political ideology. Those with a conservative ideology have a systematically lower propensity, due in part to their lower levels of trust in science. This is consistent with the previous findings regarding participation in a hypothetical AD cohort study^18^. But we show that willingness to participate in such an AD study is but one manifestation of—and not a particularly strong proxy for–this general propensity. These finding raise important issues for the recruitment and retention of subjects for medical studies and the generalizability of results from such studies.

The generalizability of results from clinical trials and other long-term observational studies depends on the inclusion of diverse pools of subjects that are representative of the population. One important area of concern is the historic and continued under-representation of minorities^30,31^. An important reason for the low participation rates of minorities is their mis-trust of medical researchers^32,33^. Our findings confirm this. The results in Table 2 show that African American respondents had significantly lower confidence in science. Also, African Americans—and to a lesser extent, Hispanics–had a lower propensity to participate in medical research.

Our results also highlight an additional threat to the generalizability of medical studies. The ideological divide in participation in medical research suggests that clinical trials and other long-term observational studies likely over-represent those with a liberal political ideology. This ideological bias in participants is potentially consequential to the quality of these studies because significant health conditions and behaviors differ with political ideology. Relative to liberals, conservatives report better physical health, are less likely to drink excessively or smoke, have poorer diets, and have lower mortality rates^12–14^. To the extent these health behaviors and conditions are relevant to a medical study, the interpretation of results and the recruitment of participants should attend to the ideological difference in participation.

This ideological divide in participation might be reduced in at least two ways. First, medical research activities can be designed to reduce the level of trust required of participants by increasing transparency^34^. For example, Americans were more willing to participate in hypothetical long-term medical studies if their research results were returned to them^8,18^. Second, the medical and scientific communities can work to increase confidence in science. Americans’ ideological commitments are fairly stable and difficult to manipulate^6,26,35^. But the ideological connection to confidence in the scientific community is, at least in part, responsive to how medical science is framed^36^. The presentation of scientific information and medical research to conservatives in a way that is consistent—or at least does not challenge—with their ideological commitments may raise their confidence in science^36–38^.

One limitation of this study is its focus on older Americans. The older subset is the pertinent population for many medical studies, particularly clinical trials and long-term observational studies. Thus, understanding participation in that group is important. Nevertheless, a complete picture of how the ideological divide among Americans affect participation includes attention to younger Americans. Indeed, the National Institution of Health recently emphasized the importance of including all ages—from children to the elderly—in clinical trials^39^.

## Methods

### Participants

The required sample size for an alpha of 0.05 and a power of 0.80 for a Baron-Kenny test of partial mediation with small effects (0.14) of ideology on confidence in science and ideology on propensity to participate (both direct and indirect) is 562 survey respondents^40^. We selected small effect sizes to be conservative in our power calculation, although findings of past studies suggest a moderate effect of ideology on confidence in science and on related behavior^1,29,41^. The TAPS survey participants [N = 1592] in September 2015 included 1359 respondents who participated in the July 2014 TAPS survey. Of the 1592 respondents, 1132 were age 45 or older, which is the age-based subset of interest in our study. Informed consent was obtained from all subjects.

The survey questions used to measure respondents’ willingness to participate in a hypothetical AD study and respondents’ confidence in institutions—including the scientific community—were only asked in the 2014 survey. This created missing values on those variables for 15% of respondents in the 2015 survey. The reported results of the IRT model in Figs. 1 and 2 and the regression models in Table 2 excluded the respondents with missing data. To ensure that missing data did not influence the results, we re-estimated the mediation analysis with the full information maximum likelihood (FIML) method. Compared to common solutions for missing data, FIML provides relatively unbiased and efficient estimates^42^. The regression results based on FIML are very similar to those from the mediation analysis with the missing data excluded **(**see Supplementary Table S1 online).

The TAPS survey includes sampling weights designed to adjust the data to be representative of the US adult population^43^. The weights were designed to match the adult American population by, in part, applying low weights to older respondents. Thus, these weights are less appropriate for the sample of interest in this study: Americans at least 45 years of age. Nevertheless, where possible we checked the robustness of our results to the introduction of sampling weights. Sampling weighted data support the same empirical conclusions as those described above for the IRT model and the mediation analysis (see Supplementary Table S1 online)^44^.

### Coding of variables

*Participated in clinical trial*: Respondents were asked “Have you ever participated as a subject in a clinical trial for a drug?” (yes, no, don’t know). We eliminated the 6 respondents who refused to answer. The 6 per cent of responses of “yes” were coded (1). The 91 per cent of responses of “no” and 3 percent of responses of “don’t know” were coded as (0).

*Participated in long-term medical study*: Respondents were asked “Have you ever participated as a subject in a long-term medical research study involving regular medical evaluations?” (yes, longer than 10 years; yes, longer than 2 years; yes, less than 2 years; no). We eliminated the 6 respondents who refused to answer. The 8 per cent of responses indicating “yes” and any length of time were coded (1). The 92 per cent of responses of “no” were coded (0).

*Participated in fundraiser*: Respondents were asked “Have you ever participated in a fundraiser for medical research (e.g., run in a race to benefit cancer)?” (yes, no, don’t know). We eliminated the 6 respondents who refused to answer. The 49 per cent of responses of “yes” were coded (1). The 49 per cent of responses of “no” and 2 per cent of responses of “don’t know” were coded (0).

*Donated blood*: Respondents were asked “Have you ever donated blood?” (yes, no, don’t know). The 65 per cent of responses of “yes” were coded (1). The 34 per cent of responses of “no” and 1 per cent of responses of “don’t now” were coded (0).

*Willing to donate organ*: Respondents were asked “Would you be willing to donate one of your organs to an organ donation service immediately after your death?” (yes, no, don’t know). We eliminated the 4 respondents who refused to answer. The 71 per cent of responses of “yes” were coded (1). The 14 per cent of responses of “no” and the 15 per cent of responses of “don't know” were coded as (0).

*Willing to participate in Alzheimer Disease (AD) study*: As in Gabel et al.^18^,  survey respondents were presented with the following vignette describing the research study:

Some healthy people join research studies about Alzheimer’s Disease. Such studies often involve a 2-3 h interview at a medical center where the person tells their medical history, completes tests of memory and thinking, and has a physical examination. These interviews and test are repeated every year, for 10 or more years. Information from these and other tests, such as a brain scan (MRI) and a spinal tap, help researchers better understand Alzheimer’s Disease so that better treatments can be developed. The test results are kept confidential and are not typically shared with the study participants.

Respondents were asked to indicate whether they agreed or disagreed with the following statement: “I would be willing to join such a study about Alzheimer’s Disease.” The response options were on a 5-point likert scale: strongly agree, agree, neutral, disagree, strongly disagree. We eliminated the 8 respondents who refused to answer. The 17 per cent of the responses of “strongly agree” were coded as (1). All other responses were coded as (0).

*Political ideology*: As in Gabel et al.^18^, the TAPS survey asked respondents to place themselves on a 7-point ideological Likert scale. The question read, “In terms of your political views, do you think of yourself as:” (very conservative, conservative, slightly conservative, moderate, slightly liberal, liberal, very liberal) and “don’t know”. The 95 respondents who chose “don’t know” or “moderate” received a follow-up question, “If you had to choose, would you consider yourself a liberal or conservative?” Responses of “liberal” or “conservative” were classified as “slightly liberal” or “slightly conservative” on the original scale. We dropped from the analysis the 16 respondents who refused to answer. Finally, we recoded the responses so that the variable used in the analyses ranges from 0 (very conservative) to 6 (very liberal).

*Confidence in science*: Respondents were asked “Indicate how much confidence you have in the people running each of the following: [scientific community].” The response options were on a 4-point Likert response scale: great deal of confidence, quite a lot of confidence, only some confidence, very little confidence. We eliminated the 8 respondents who refused to answer. The responses were recoded so that the variable ranges from 0 (very little confidence) to 3 (great deal of confidence).

*General confidence in institutions*: Along with the question regarding confidence in the scientific community, the survey included a set of questions about confidence in other institutions: the press, television news, the Supreme Court, Congress, and the military. We estimated a one-factor model of responses to these questions. All items were positively related to the single factor and the eigenvalues (factor 1 = 1.81; factor 2 = 0.36) strongly supported a single factor. We then generated a factor score for each respondent using regression factor scoring. We eliminated the 29 respondents who refused to answer at least one of these.

*Age*: Respondents were assigned to one of four categories according to years of age: 1 (18–29); 2 (30–44); 3 (45–59); 4 (60 or older).

*African American*: Self-identified African Americans were coded (1), all others were coded (0).

*Hispanic*: Self-identified Hispanics were coded (1), all others were coded (0).

*Female*: Self-identified females were coded (1), all others were coded (0).

*Education*: Respondents were asked “What is the highest level of school you have completed?” and chose from fifteen ordinal options ranging from “no formal education” (1) to “Doctorate degree” (15). We eliminated the 7 respondents who refused to answer.

*Metropolitan resident*: Residents of a metropolitan statistical area were coded (1), all other were coded (0).

### Statistical analysis

All statistical analyses were conducted with STATA version 15.1. The factor analyses were conducted with the principal factor method and the “polychoric” command, which generates a correlation matrix based on dichotomous variables. The IRT models were estimated with the “irt” command and the relevant models for one and two parameters. We estimated both a one and a two-parameter IRT model. A likelihood ratio test strongly supports the two-parameter model (*p* < 0.01), where each item has its own discrimination parameter. The mediation analysis was conducted with the “medeff” command^45,46^. This command provides estimates of mediated effects with robust (Huber/White/sandwich) standard errors. To account for missing data, we used the “sem” command with the “mlmv” option to estimate full information maximum likelihood (FIML) linear regressions reported in Supplementary Table S1 online.

The mediation analyses included controls for potentially confounding factors. We controlled for respondent’s general confidence in institutions to ensure that our measure of confidence in the scientific community was not simply capturing respondents’ general antipathy or support for social institutions. We controlled for gender, years of education, African American ethnicity and Hispanic ethnicity because all of these characteristics have been shown to correlate with political ideology and may influence propensity to participate because they affect volunteerism, trust in medicine, or familiarity with medical studies^33,47–50^. We also controlled for age and for residence in a metropolitan area, which may influence a respondent’s opportunity to participate in medical research activities.

## Supplementary Information

Supplementary Information.

## Data Availability

The dataset and code for analysis are available from the author upon request.
