# Supplementary material for: The ideological divide in confidence in science and participation in medical research
Source: Sci Rep. 2021 Feb 4;11:3120. doi: 10.1038/s41598-021-82516-6 (PMC7862386; doi:10.1038/s41598-021-82516-6)
Supplement: Supplementary file 1 — Supplementary Information. [file 41598_2021_82516_MOESM1_ESM.docx]

**Supplementary materials for**

**The Ideological Divide in Confidence in Science and Participation in Medical Research**

**Authors**

Matthew Gabel, PhD (Washington University in St. Louis)

Jonathan Gooblar, PhD (Stanford University School of Medicine)

Catherine M. Roe, PhD (Washington University in St. Louis)

John C. Morris, MD (Washington University in St. Louis)

**Supplementary Table S1.** Robustness tests for regression results for mediation analysis based on subset of respondents at least 45 years old. Each column presents the ordinary least squares unstandardized regression coefficients, with robust standard errors in parentheses. Column (1) corresponds to equation (1) of the mediation analysis (*Confidence in Science* is the dependent variable). Column (2) corresponds to equation (2) in the mediation analysis (*Propensity to Participa*te is the dependent variable). **p* < .05; ***p* < .01, two-tailed test.

|  | **Ordinary Least Squares**  **with Sampling Weights** | | **Full information Maximum Likelihood (FIML)** | | **FIML with**  **Sampling Weights** | |
| --- | --- | --- | --- | --- | --- | --- |
|  | (1) | (2) | (1) | (2) | (1) | (2) |
| **Political Ideology** | 0.128** | 0.043* | 0.093** | 0.029* | 0.128** | 0.042* |
|  | (0.026) | (0.019) | (0.013) | (0.013) | (0.026) | (0.019) |
| **Confidence in Science** | - | 0.094* | - | 0.086** | - | 0.091* |
|  |  | (0.047) |  | (0.030) |  | (0.046) |
| **Confidence in Institutions** | 0.342** | 0.003 | 0.354** | 0.013 | 0.347** | 0.012 |
|  | (0.047) | (0.040) | (0.026) | (0.027) | (0.046) | (0.036) |
| **Age** | 0.204** | 0.027 | 0.161** | 0.092* | 0.212** | 0.019 |
|  | (0.080) | (0.060) | (0.045) | (0.042) | (0.078) | (0.059) |
| **Female** | 0.083 | 0.057 | 0.184** | 0.029 | 0.106 | 0.043 |
|  | (0.080) | (0.059) | (0.045) | (0.042) | (0.077) | (0.058) |
| **Education** | 0.060** | 0.055** | 0.083** | 0.058** | 0.061** | 0.056** |
|  | (0.023) | (0.013) | (0.012) | (0.012) | (0.023) | (0.013) |
| **African American** | -0.420* | -0.397** | -0.367** | -0.264** | -0.404* | -0.392** |
|  | (0.210) | (0.140) | (0.090) | (0.084) | (0.204) | (0.136) |
| **Hispanic** | -0.012 | -0.274* | -0.146 | -0.120 | -0.014 | -0.276* |
|  | (0.148) | (0.110) | (0.090) | (0.084) | (0.145) | (0.110) |
| **Metropolitan resident** | 0.199* | 0.012 | 0.191** | 0.121* | 0.213* | 0.015 |
|  | (0.093) | (0.075) | (0.063) | (0.058) | (0.090) | (0.072) |
| **R^2^** | 0.31 | 0.14 | - | - | - | - |
| **N** | 932 | | 1123 | | 1123 | |
